# Impact of a Free Influenza Vaccination Policy on Older Adults in Zhejiang, China: Cross-Sectional Survey of Vaccination Willingness and Determinants

**DOI:** 10.2196/73940

**Published:** 2025-09-15

**Authors:** Yusui Zhao, Jinhang Xu, Xuehai Zhang, Yue Xu, Xiaotong Yan, Shaofan Ren, Lixin Wang

**Affiliations:** 1 Zhejiang Provincial Center for Disease Control and Prevention, 3399 Binsheng Road, Hangzhou, 310051, China, 86 0571-87115111

**Keywords:** influenza vaccine, vaccination willingness, older adults, health policy, China

## Abstract

**Background:**

In 2024, Zhejiang Province introduced a new policy offering free influenza vaccinations to individuals aged 60 years and older. However, the vaccination willingness among the newly eligible 60-69 years age group remains ambiguous in comparison to those aged 70 years and older.

**Objective:**

This study aimed to evaluate the willingness of individuals aged ≥60 years in Zhejiang Province, China, to receive free influenza vaccines under a newly implemented policy. It further explored their sources of influenza-related health information and identified key determinants of vaccination hesitancy across age subgroups.

**Methods:**

A cross-sectional survey was conducted using multistage convenience sampling via on-site questionnaires. Structured questionnaires were administered to 7162 eligible participants aged ≥60 years from March to May 2024. Valid responses (n=7103; response rate: 99.18%) were analyzed via logistic regression and Kruskal-Wallis tests.

**Results:**

Overall vaccination willingness was 73.15% (5196/7103), with 11.71% (832/7103) refusal and 15.14% (1075/7103) hesitancy. Key predictors of hesitancy included male gender (odds ratio [OR] 1.27, 95% CI 1.05‐1.54), ages 60‐69 years (OR 1.46, 95% CI 1.06‐2.02), corporate employment (OR 0.75, 95% CI 0.58‐0.98), and absence of chronic diseases (OR 2.06, 95% CI 1.44‐2.96). The 60‐69 year age group demonstrated lower awareness of the free policy (61.9% vs 73.72% in the ≥70 years group; H=61.25, *P*<.001) but higher engagement with social media (WeChat [Tencent Holdings Limited]: H=345.44; TikTok [ByteDance Ltd]: H=294.66; *P*<.001) for health information.

**Conclusions:**

Despite high willingness, knowledge gaps persist, particularly among adults aged 60‐69 years. Targeted dissemination of policy information via social media platforms (eg, WeChat and TikTok) and community-driven campaigns is recommended to enhance vaccination uptake. This approach may serve as a model for regions implementing similar policies. Future studies should track actual vaccination uptake postpolicy and explore artificial intelligence–driven social media interventions to boost engagement.

## Introduction

Influenza, a prevalent seasonal acute respiratory infectious disease, exhibits an escalating incidence rate in China [1]. The virus is characterized by its high antigenic variability and rapid transmission, resulting in annual epidemics. Globally, influenza is responsible for approximately 390,000 deaths annually, with two-thirds of these fatalities occurring among older adults [2]. In China, individuals aged ≥60 years account for 80% of the nation’s influenza-associated excess respiratory mortality [3]. Within Zhejiang Province, surveillance data from 2009 to 2021 indicate 990,016 reported cases and 8 deaths, with estimated actual cases 12.11-fold higher than reported [4]. Influenza vaccination—recognized as the most cost-effective intervention for preventing respiratory infections by the Chinese Center for Disease Control and Prevention [5]—reduces disease burden, hospitalizations, and mortality in older adults [6,7]. A 2013 meta-analysis of 95 studies confirmed vaccination prevents 28% of complications, 39% of influenza-like illnesses, and 49% of confirmed cases in this demographic [8].

The influenza vaccination uptake among the older population in China has traditionally been low. For instance, during the 2011‐2012 flu season, only 4.3% of individuals aged ≥60 years in 9 Chinese cities were vaccinated against influenza [9]. This rate further declined to 3.8% in the 2014‐2015 flu season [10]. However, in recent years, there has been a marked increase in the vaccination rate among older adults, attributable to the Chinese government’s heightened emphasis on vaccinations for older adults. This surge is particularly noticeable in the aftermath of the COVID-19 outbreak and the introduction of free influenza vaccination policies by certain local governments. In the 2020‐2021 and 2021‐2022 periods, free influenza vaccinations were offered in 195 and 226 counties and districts, respectively, leading to vaccination rates of 45.7% and 32.9% among the older populations in these regions [11]. Despite this progress, the influenza vaccination rate among older adults in China remains below the World Health Organization’s target of 75%.

In China, influenza vaccines were categorized as Category II, necessitating voluntary self-funded vaccination by residents [12]. This classification contributed to the low uptake of influenza vaccination. However, with China’s economic progression, policies granting free influenza vaccinations to individuals aged ≥60 years were first introduced in major cities, including Beijing, Shanghai, and Shenzhen [13-15]. Extensive research confirmed that these measures substantially elevated both the willingness and rate of influenza vaccination among older adults. As a result, the initiative extended to other regions, encompassing areas within Zhejiang and Shandong provinces [16,17]. Notably, Zhejiang Province was among the pioneers in this endeavor. Specific counties and districts, such as Taizhou and Ningbo [18,19], began offering complimentary influenza vaccinations to those aged ≥60 years from 2017, aligning with local government initiatives for public welfare. By 2020, Zhejiang had fully adopted the policy for residents aged ≥70 years [16], and by 2024, it was expanded to cover all residents aged ≥60 years in the province [20].

Several studies have examined influenza vaccination willingness in Chinese regions with similar policies. In Beijing (free for ≥60 years since 2007), willingness reached 76.61% post policy implementation [21]. Shanghai reported 80.6% willingness among adults ≥50 years [22], while Hangzhou (Zhejiang’s capital) observed 85.98% uptake in the ≥70 year age group under free vaccination [16]. Lower willingness was noted in non–first-tier cities (eg, 60.7% in Chongqing [23] and 53.66% in Kaifu District, Changsha [24]). These studies consistently identified policy awareness, income level, and prior vaccination history as key determinants. However, no prior study has assessed the newly eligible 60‐69 year age group in a province-wide free vaccination program, highlighting the necessity of this investigation.

Despite the existing circumstances, the influenza vaccination rate among individuals aged ≥60 years in Zhejiang Province was a mere 21.76% [25] prior to the introduction of the universal free influenza vaccination policy for this age group. The potential impact of this policy on vaccination rates, particularly among those aged between 60 and 69 years old, remains uncertain. However, an individual’s willingness to receive the flu vaccine is a critical factor influencing vaccination rates; the stronger the willingness, the higher the likelihood of actual vaccination [26,27]. Given this context, our study aims to (1) analyze the willingness to receive the influenza vaccine and its determinants among individuals aged ≥60 years in Zhejiang Province under the free vaccination policy in 2024, (2) investigate the ways from which different age groups of older individuals acquire knowledge about influenza prevention and treatment, and (3) identify the primary reasons why different age groups of older individuals are willing or unwilling to get vaccinated against influenza.

## Methods

### Study Setting, Sampling and Recruitment Procedure

Zhejiang was a province located in the southeastern part of China with a population of 66 million at the end of 2023. Among this population, approximately 14 million individuals were aged ≥60 years, representing about 21.5% of the total population. In addition, Zhejiang had experienced the second-largest influx of migrant workers in China and remained a significant province for this demographic group. This situation further increased the risk of infectious disease transmission among them.

The policy was announced via government portals in January 2024. After 2 months for policy dissemination, a questionnaire survey was administered from March to May 2024 to the selected participants. Inclusion criteria were as follows: (1) aged ≥60 years, (2) residents of the selected community or village, and (3) individuals who were informed about the study’s content and purpose and voluntarily agreed to participate. Exclusion criteria included (1) plans to be away from the community or village for more than 3 months in the upcoming year, (2) cognitive impairments that hindered normal communication, and (3) lack of cooperation with the study and poor compliance (Multimedia Appendix 1).

### Measures

The instrument was developed based on the World Health Organization flu vaccine hesitancy scales. Cognitive testing was conducted with 20 older participants in January 2024, followed by a pilot survey in 2 communities (n=200). Cronbach α improved from 0.68 to 0.765 after revising items with poor discrimination (item-total correlation <0.2).

The self-developed questionnaire was composed of 3 different parts, including sociodemographic information, knowledge on influenza prevention and treatment, and reasons for willingness or unwillingness to receive influenza vaccination (Multimedia Appendix 2).

Sex, age, ethnicity, marital status, family structure, education, occupation, income, chronic illness status, and history of influenza vaccination were included in sociodemographic information.

The section of knowledge on influenza prevention and treatment consisted of 11 items, segmented into 3 categories: basic knowledge of influenza, knowledge on preventing influenza, and understanding of influenza vaccination. The fundamental knowledge contained 7 single-choice questions. The influenza prevention knowledge had 1 multiple-choice question, while the influenza vaccination knowledge comprised 3 questions, with 2 being single-choice and 1 a multiple-choice. Each correct answer in the single-choice section was allocated 1 point, whereas a completely accurate response in the multiple-choice section was given 2 points. Incorrect answers, whether due to overselection, underselection, incorrect selection, or omission, were not awarded any points. The maximum possible score was 13, known as the influenza prevention knowledge score. Each of the 7 items had a content validity index greater than 0.8 and the overall Cronbach α coefficient was 0.765. The Kaiser-Meyer-Olkin measure was 0.855 and the Bartlett test *P* value was less than .01, indicating adequate reliability and validity.

The section of reasons for willingness or unwillingness to receive influenza vaccination consisted of 3 questions: “How do you learn about health information?,” “Why are you willing to get a flu vaccine?,” and “Why haven’t you received the flu vaccine?” The 3 questions mentioned above were all presented in a multiple-choice format for respondents.

The following were also taken into consideration during data collection:

- Occupation: for older respondents, if they are retired, record the occupation they were engaged in before retirement. If they are still working, record their current occupation.
- Income: personal monthly income refers to the total amount of taxable income earned by an individual within a tax month. For older respondents, sources of income include both wages (or pensions) and other types of income combined.
- Chronic diseases refer to conditions that have been definitively diagnosed by a physician, including hypertension, diabetes mellitus, hyperlipidemia, chronic obstructive pulmonary disease, bronchitis, stroke, coronary artery disease, and tumors.

### Data Analysis

A database was constructed using EpiData 3.1 software (EpiData Association), with data entry executed in duplicate to ensure accuracy. Statistical analysis was performed using SAS Viya Long-Term Support 2024.03 (SAS Institute). Descriptive analysis was used to examine demographic characteristics, and the Kruskal-Wallis (H) test was used for this purpose. ANOVA (*F* test) was conducted to compare scores related to influenza prevention and treatment knowledge. Multivariate logistic regression analysis was implemented to identify factors that influence vaccination intention. A *P* value less than .05 was deemed statistically significant.

The dependent variable is influenza vaccination willingness (positive=1; hesitation=2; negative=3). The term “positive” is used to denote survey participants who explicitly stated their willingness to receive the influenza vaccine. The term “hesitation” refers to those who demonstrated hesitancy, while “negative” pertains to individuals who clearly expressed their unwillingness to receive the vaccine. The independent variables consistently included in all models are as follows: sex (male=1; female=2), age (60‐69 years old=1; 70‐79 years old=2; aged ≥80 years old=3), marital status (unmarried=1; married=2; unmarried or divorced or widowed=3), family structure (solitary living=1; living with spouse or children=2; living with spouse and children=3; other=4), education level (primary school or lower=1; middle school=2; high school or technical school=3; college or higher=4), occupation (agency or institutional personnel=1; medical staff=2; farmers corporate staff=3; sole proprietors=4; other=5), chronic illness status (none=1; 1 to 2=2; 3 or more=3), history of influenza vaccination (yes=1; no=2), and income (less than 2000 RMB [approximately US $281]=1; 2000‐4999 RMB [approximately US $281-$703]=2; 5000‐10,000 RMB [approximately US $703-$1407]=3; more than 10,000 RMB [approximately >US $1407]=4; unclear=5). All conversions from RMB to US $ are based on the average exchange rate on March 1, 2024 (US $1=7.1066 RMB).

Ethical Considerations

The study was conducted in accordance with the Declaration of Helsinki. Informed consent was obtained from all participants or their guardians. Each participant received a small gift valued at 100 RMB (approximately US $14) as compensation. Surveyors recorded responses without personal identifiers; data were anonymized during electronic entry using coded IDs. The study protocol was approved by the ethics committee of the Zhejiang Provincial Center for Disease Control and Prevention (2022-016-02).

## Results

### Demographics and Univariate Analysis of Vaccination Willingness

In 2024, a total of 7162 individuals were surveyed, yielding 7103 valid responses, reflecting a response rate of 99.18%. The gender distribution was nearly equal, with 3566 males and 3537 females. Approximately 91.9% (6533/7103) of the participants were between the ages of 60 to 79 years, while only 8.0% (570/7103) were aged 80 years or older. Over half of the respondents (4577/7103, 64.4%) had an educational level at or below primary school, and more than 80% (5969/7103) reported an average monthly income of less than 5000 RMB (approximately US $703).

The univariate analysis results revealed that factors such as sex, age, marital status, family structure, education, occupation, income, chronic illness status, and influenza vaccination history significantly influenced respondents’ willingness to receive the influenza vaccine. Of these, the most influential factor was found to be influenza vaccination history. Respondents with a history of influenza vaccination demonstrated a higher willingness to be vaccinated compared to those who had never been vaccinated (n=3572, 86.7% vs n=1624, 54.5%) against influenza. Age was identified as the second most influential factor. Respondents aged 70‐79 years expressed the highest willingness to receive the vaccine (n=2389, 78.1%), while those aged 60‐69 years showed the least willingness (n=2371, 68.3%). Medical staff showed the highest willingness to be vaccinated compared to other occupational groups (n=176, 81.9%). Furthermore, respondents with chronic diseases were more likely to express a willingness to receive the influenza vaccine, although they also had a higher proportion of unwillingness to be vaccinated (Table S1 in Multimedia Appendix 3).

### Multivariate Analysis of Vaccination Willingness

A multivariate logistic regression analysis was performed, using the willingness to receive the influenza vaccine as the dependent variable and indicators that demonstrated statistically significant differences in univariate analysis as independent variables. The findings revealed that the 60‐69 year age group, medical staff, individuals with chronic illnesses, and those who had a history of influenza vaccination were all contributing factors to the willingness to receive the influenza vaccine. Notably, residents with a history of influenza vaccination were 7.82 times more likely to express willingness to receive the influenza vaccine compared to those without such a history (odds ratio [OR] 7.82, 95% CI 6.52‐9.39; *P*<.001) (Table 1).

**Table 1.** The multivariate logistic regression results of factors associated with influenza vaccination willingness of participants.

Dependent and independent variable level		B	SE	Wald *χ*^2^ (*df*)	*P* value	OR^a^ (95% CI)
Positive						
	Age (years)					
	60‐69	0.38	0.17	5.36 (1)	.02	1.46 (1.06‐2.02)
	70‐79	0.06	0.16	0.15 (1)	.70	1.84 (1.40‐2.41)
	≥80 (Reference)	—^b^	—	—	—	—
	Occupation					
	Agency or Institutional personnel	0.06	0.24	0.07 (1)	.79	1.07 (0.67‐1.69)
	Medical staff	0.11	0.34	0.11 (1)	.74	1.12 (0.58‐2.18)
	Corporate staff	–0.28	0.13	4.45 (1)	.04	0.75 (0.58‐0.98)
	Sole proprietors	–0.22	0.14	2.74 (1)	.10	0.80 (0.62‐1.04)
	Farmers (Reference)	—	—	—	—	—
	Chronic illness status					
	None	0.37	0.15	6.12 (1)	.01	1.44 (1.08‐1.93)
	1 to 2	0.31	0.13	5.42 (1)	.02	1.37 (1.05‐1.77)
	3 or more (Reference)	—	—	—	—	—
	Influenza vaccination history					
	No	2.06	0.09	490.76 (1)	<.001	7.82 (6.52‐9.39)
	Yes (Reference)	—	—	—	—	—
Hesitation						
	Sex					
	Male	0.24	0.10	6.07 (1)	.01	1.27 (1.05‐1.54)
	Female (Reference)	—	—	—	—	—
	Influenza vaccination history					
	No	0.57	0.11	25.76 (1)	<.001	1.76 (1.42‐2.19)
	Yes (Reference)	—	—	—	—	—
	Chronic illness status					
	None	0.72	0.18	15.51 (1)	<.001	2.06 (1.44‐2.96)
	1 to 2	0.39	0.17	5.15 (1)	.02	1.47 (1.05‐2.06)
	3 or more (Reference)	—	—	—	—	—

a OR: odds ratio.

b Not applicable.

### Comparison of Influenza Prevention and Treatment Knowledge Scores

The results showed that the scores for influenza prevention and treatment knowledge among respondents aged 60‐69 years was 6.5 (SD 3.19); among those aged 70‐79 years, it was 6.1 (SD 3.24), and among those aged ≥80 years, it was 5.2 (SD 3.37). There was a statistically significant difference in the scores of influenza prevention and treatment knowledge among different age groups (*F*_2_=45.55, *P*<.001), with older age groups having lower scores.

### Access to Health Information

The findings indicated that television, doctors, and family members were the main ways of obtaining health knowledge. Compared to the 70‐79 and ≥80 year groups, individuals in the 60‐69 year age group exhibited a higher propensity for leveraging new media platforms such as WeChat (Tencent Holdings Limited; H=345.44, *P*<.001) and TikTok (ByteDance Ltd; H=294.66, *P*<.001) as primary ways for acquiring health information. Despite high social media use (WeChat: n=1366, 39.34%), the 60‐69 year group’s policy awareness showed no correlation with platform engagement (*r*=0.04, *P*=.11), suggesting ineffective information diffusion (Table S4 in Multimedia Appendix 4).

### Reasons for Willingness to Receive Influenza Vaccination

A questionnaire survey was conducted among respondents who were willing to receive the influenza vaccine. The findings indicated that the predominant reason influencing participants’ willingness to receive the influenza vaccine was “awareness of the free vaccine policy.” Awareness of the free policy was 70.16% (2013/2869) overall but significantly lower in those aged 60‐69 years (n=867, 61.9% vs n=2002, 73.72% in ≥70 year group; *P*<.001). In addition, statistically significant differences among different age groups were noted for the reasons “active promotion by the community” (H=6.740, *P*=.03) and “awareness of the advantages of getting vaccinated” (H=33.125, *P*<.001). No other reasons demonstrated a statistically significant difference (Table S5 in Multimedia Appendix 5).

### Reasons for Unwillingness to Receive Influenza Vaccination

The results indicated that “concerns about the necessity of vaccination,” “not previously acknowledged or recognized,” and “concerns about the effectiveness of vaccines” were the 3 primary reasons for respondents not willing to receive the influenza vaccine. These reasons did not show any statistically significant differences among different age groups. In comparison, respondents in the ≥80 year group were more concerned about the safety of the vaccine (H=10.96, *P*=.004). Those in the 70‐79 year age group were more likely to refuse the vaccine due to contraindications (H=28.40, *P*<.001), while those in the 60‐69 year age group were more concerned about the burden of vaccination (H=6.96, *P*=.03) (Table S6 in Multimedia Appendix 6).

## Discussion

### Principal Findings

This study indicated that the willingness to receive the influenza vaccine among residents aged ≥60 years in Zhejiang Province was 73.15%, which was lower than the vaccination willingness rates among the older population in first-tier Chinese cities such as Beijing and Shanghai [21,22]. It was also lower than that of Hangzhou, the capital city of Zhejiang Province, but higher than that of other non–first-tier cities in China [23,24], as well as higher than similar survey results from countries such as the United States and Italy [28,29]. On one hand, after 3 years of the COVID-19 pandemic, China had unprecedentedly intensified its publicity efforts on respiratory infectious disease prevention, significantly improving residents’ knowledge, attitudes, and behaviors regarding respiratory infectious disease control [30]. On the other hand, the economic burden of influenza vaccination affected residents’ willingness to get vaccinated, especially for low-income middle-aged and older individuals. Whether the vaccine was free played a crucial role in their decision to get vaccinated [31].

This study found that older individuals who were female, aged ≥70 years, had a history of influenza vaccination, and had chronic diseases were more willing to receive the flu vaccine. This finding was consistent with some domestic studies [32,33]. The history of influenza vaccination was the most significant factor influencing the willingness to vaccinate among the older adults [26,34,35]. The willingness to vaccinate among the older adults who had previously received the influenza vaccine was 7.823 times higher than that of those who had not. Prior recipients may perceive reduced infection severity, but causality between vaccination history and economic burden relief remains unproven. Age was another important factor. Compared to those aged ≥70 years, the willingness to receive the vaccine was lowest among those aged 60‐69 years, who also had the highest proportion of hesitation responses. The primary reason was likely due to the recent implementation of a free influenza vaccination policy for this age group in Zhejiang Province, which many were still unaware of. The free vaccination policy was one of the most crucial factors driving vaccination rates, especially among low-income older populations [36]. When examining reasons for willingness to vaccinate, it was found that the proportion of those aware of the free vaccine policy in the 60‐69 year age group was significantly lower than in other age groups, further explaining their lower vaccination willingness. However, encouragingly, the 60‐69 year age group had a higher acceptance of the influenza vaccine and better recognized its benefits.

To assess knowledge levels about influenza prevention and treatment across different age groups, an 11-question questionnaire was designed. Analysis showed that the 60‐69 year age group scored highest on the knowledge assessment, yet they had the lowest willingness to vaccinate, contradicting previous study in Hangzhou [16]. This discrepancy might be attributed to the limited number of questions in their questionnaire (consisting of merely 5 questions), which could introduce randomness. To ensure the questionnaire’s accuracy and reliability, it was repeatedly revised until both its reliability and validity were satisfactory. Another analysis revealed that despite higher educational levels, better mental health, and stronger memory among the 60‐69 year age group, their unfamiliarity with the newly introduced free vaccination policy contributed to their lower vaccination willingness compared to those aged ≥70 years.

This study found that television, medical staff, and family members are the main ways for older adults to obtain health information. In China, traditional media such as television remains the primary source of health information for older adults [16]. Despite this, their trust in doctors is higher [37]. It is recommended that medical staff should be encouraged to strengthen the promotion of influenza vaccine policy and related prevention knowledge among older adults, in order to better help them establish a positive willingness to get vaccinated against the flu and thus make the decision to receive the influenza vaccine. What is different is that compared to those aged ≥70 years, the 60‐69 year age group more often obtains health information through television, while the ≥80 year age group more often relies on family members for health information. Therefore, for the newly implemented policy in Zhejiang Province of free influenza vaccination for those aged 60‐69 years, it is advisable to promote this policy to the older adults through local mainstream television media. This would not only increase the coverage of the promotion but also enhance its authority and credibility. It is worth noting that with the widespread proliferation of the internet in China, new media represented by WeChat and TikTok have risen strongly. According to statistics, from 2010 to 2020, over a 10-year period, the number of internet users aged ≥60 years increased from 8.67 million to 60.54 million, with the proportion of older internet users in the older population increasing from 4.9% to 23.8% [38]. Compared to those aged ≥70 years, the 60‐69 year age group is far more active on WeChat or TikTok every day. This also suggests that for the 60‐69 year age group, the way of obtaining health information is gradually changing, and new media is playing an increasingly important role.

From the analysis of reasons for willingness to receive influenza vaccination, apart from “awareness of the free vaccine policy,” “active promotion by the community” also plays a crucial role in encouraging older individuals to get vaccinated. Compared to those aged ≥70 years, individuals in the 60‐69 year age group are more likely to believe that receiving the influenza vaccine can benefit their health. They also pay more attention to the necessity of getting vaccinated [39]. On the other hand, those aged ≥70 years are more concerned about the safety of the influenza vaccine [40]. This highlights the importance of enhancing community promotion of the free influenza vaccination policy, as well as disseminating information about the necessity and safety of the influenza vaccine, to increase the willingness to vaccinate among older adults, particularly those in the 60‐69 year age group. Future implementation research should examine digital outreach efficacy through platforms such as TikTok or WeChat, particularly in low-resource settings.

This study has some limitations. First, the sampling method used in this study was a multistage convenience sampling approach to obtain respondents. The selection process favors individuals more likely to be accessible or compliant, skewing the results toward higher vaccination willingness. Second, the investigators were generally doctors from local hospitals or community health service centers (township health clinics), who were relatively familiar with the respondents. During the survey process, respondents might tend to provide more positive responses, which could potentially overestimate their willingness to receive influenza vaccination. Third, some questions were answered based on recall alone, such as influenza vaccination history and chronic illness status, which might lead to recall bias. Finally, the cross-sectional survey method used in this study might affect the generalizability of the results.

### Conclusions

Targeted dissemination via social media (eg, TikTok or WeChat) for the 60‐69 year age group and community-driven policy education are critical next steps. This approach may serve as a model for regions implementing similar policies. Future studies should track actual vaccination uptake and test artificial intelligence–optimized messaging through digital platforms.

## Supplementary material

10.2196/73940 Multimedia Appendix 1 Sampling framework and sample size calculation.

10.2196/73940 Multimedia Appendix 2 Questionnaire on influenza vaccination willingness among the older population in Zhejiang Province.

10.2196/73940 Multimedia Appendix 3 Comparison of influenza vaccination willingness among different groups.

10.2196/73940 Multimedia Appendix 4 The means of obtaining health information among different age groups of participants.

10.2196/73940 Multimedia Appendix 5 Comparison of the reasons for willingness to receive influenza vaccination among different age groups.

10.2196/73940 Multimedia Appendix 6 Comparison of the reasons for unwillingness to receive influenza vaccination among different age groups.

## Abbreviations

OR: odds ratio
