# Supplementary material for: Impact of a Free Influenza Vaccination Policy on Older Adults in Zhejiang, China: Cross-Sectional Survey of Vaccination Willingness and Determinants
Source: JMIR Hum Factors. 2025 Sep 15;12:e73940. doi: 10.2196/73940 (PMC12435753; doi:10.2196/73940)
Supplement: Multimedia Appendix 1 [file humanfactors-v12-e73940-s001.docx]

**Multimedia Appendix 1.** Sampling Framework and Sample Size Calculation Using Multi-stage Convenience Sampling in a Provincial Survey

**Sampling Framework and Sample Size Calculation**

A multi-stage convenience sampling method was utilized in this study. Initially, based on the research objectives, 35 counties were chosen randomly from a pool of 90 counties across the province. Subsequently, within each selected county, five communities or villages were identified randomly. Local hospitals and community health service centers/community health clinics employed intercept survey techniques to engage residents of these chosen communities or villages who met the study's criteria. The surveys were administered by medical professionals from these local institutions. Prior to participation, informed consent was secured from the respondents. Those who consented either completed the questionnaire independently or, if they were unable to do so, had the surveyors assist them on-site. If a potential participant declined after two notifications, their involvement was discontinued. Each survey site aimed to involve 60 residents, concluding once a sufficient number of valid questionnaires were gathered.

The sample size for each county was determined using the formula:

$$N=\frac{\mu_{\alpha}^{2}\times p(1-p)}{\delta^{2}}\times deff$$

where α (0.05) represents the significance level, µα (1.96) is the α quantile of the standard normal distribution, p (32.94%, based on the influenza vaccination rate among the elderly in China from 2021-2022) is the vaccination rate, δ (0.0824) is the maximum permissible error, and deff (1.5) is the design effect for complex sampling. After excluding invalid questionnaires and accounting for a refusal rate (20%), the final sample size for each county was approximately 200. The total sample size for all 35 counties was 7,000.
