# Supplementary material for: Impact of a Free Influenza Vaccination Policy on Older Adults in Zhejiang, China: Cross-Sectional Survey of Vaccination Willingness and Determinants
Source: JMIR Hum Factors. 2025 Sep 15;12:e73940. doi: 10.2196/73940 (PMC12435753; doi:10.2196/73940)
Supplement: Multimedia Appendix 2 [file humanfactors-v12-e73940-s002.doc]

**Multimedia Appendix 2.** Questionnaire on Influenza Vaccination Willingness among the Elderly Population in Zhejiang Province

Questionnaire on Influenza Vaccination Willingness among the Elderly Population in Zhejiang Province

coding：□□□□□□□

**Introduction and informed consent**

Hello, we are the staff of local hospitals and community health service centers/community health clinics. In order to understand the willingness of elderly population in Zhejiang Province to receive influenza vaccination, their knowledge about influenza prevention and control, and the reasons for being willing or unwilling to get vaccinated, we need to ask you a few questions, please provide some personal information, contact information, answer some questions. This information will be only to facilitate the future to find you and give you scientific health guidance. The content involved in the investigation will be kept confidential for you and will not be disclosed to any third party. So please answer truthfully. Participation in this survey will be completely voluntary, and you may refuse to answer any questions you are not willing to answer, and you can terminate the survey at any time if you wish to do so in the survey. By participating in this survey, you can acquire knowledge and information on the prevention of infectious diseases, and help relevant departments formulate strategies and measures for the prevention and control of infectious diseases. So as to effectively prevent infectious diseases and better protect everyone. The survey will take you about 15 minutes. I will give you a small souvenir after the survey. If you agree to participate in the survey, please sign and cooperate with us to complete the survey.

Thank you for your support!

**Respondent's signature after informed consent:**

**Sociodemographic characteristics**

**1. Region:**  City County (District) Community.

**2. Name**：

**3. Mobile:**

**4. Gender**：  □ male     □ female  **□**

**5. Age**：_________

**6. Ethnicity**：  □ Han □ Minority **□**

**7. Marital status**：   **□**

□Unmarried □ Married □ Separation/Divorced/Widowed

**8. Family structure：**   **□**

□ Solitary living □ Living with spouse or children

□ Living with spouse and children □ Other

**9. Education**：  **□**

□ Primary school or lower    □ Middle school

□ High school/Technical school   □ College or higher

10. **Occupation**：  **□**

□ Agency/ Institutional personnel □ Medical staff

□ Farmers  □ Corporate staff

□ Sole proprietors □ Other

**11. My monthly income：**_________¥

**12.** **Based on the doctor's diagnosis, have you ever had or currently have any of the following chronic diseases (multiple choices allowed)?**

□ Hypertension □ Diabetes Mellitus □ Hyperlipidemia

□ Chronic Obstructive Pulmonary Disease □ Bronchitis

□ Stroke Coronary □ Heart Disease □ Tumor

**Scale of influenza prevention and treatment**

**1. Have you heard of the flu?**

□ yes □ no

**2. Do you think that older adults are more susceptible to the flu?**

□ yes □ no □ Don't know

**3. Is the flu just a common cold?**

□ Yes □ No □ Not sure

**4. The flu is a respiratory disease caused by______?**

□ Bacteria □ Virus □ Air pollution □ Not sure

**5. Can the flu exacerbate underlying diseases such as hypertension and diabetes?**

□ Yes □ No □ Not sure

**6. What season is more prone to the flu?**

□ Winter and spring □ Summer and fall □ Not sure

**7. What is the best time to get a flu vaccine?**

□ 1-2 months before the peak of flu season □ During the flu season □ Not sure

**8. How often should one get a flu vaccine?**

□ Once a year □ Every 5 years □ Not sure

**9. Which of the following measures can prevent the flu? (Multiple choices allowed)**

□ Wearing a mask

□ Frequently washing hands

□ Regular ventilation

□ Healthy lifestyle including proper nutrition, regular exercise, adequate sleep

□ Vaccination

□ Don't know

**10. Do you think it is possible to receive both the flu vaccine and the pneumococcal vaccine at the same time?**

□ Yes □ No □ Not sure

**11. In which of the following situations do you think vaccination is appropriate? (Multiple choices allowed)**

□ Those with chronic underlying diseases such as hypertension and diabetes, who are stable on medication

□ Acute phase of chronic diseases such as coronary heart disease

□ Those with allergies to vaccine components or a history of severe allergies (e.g., laryngeal edema)

□ HIV carriers or infected individuals who are stably medicated □ Currently febrile

□ History of epilepsy but no seizures in the past 3 years, currently in good condition

□ Patients with Guillain-Barré syndrome

**Reasons for willingness or unwillingness to receive**

**influenza vaccination**

**1. How do you learn about health information? (Multiple choices allowed)**

**Traditional media**: □ Television □ Radio □ Newspapers/Magazines, books

**New media:** □ Websites □ WeChat □ TikTok

**Interpersonal communication:** □ Doctors □ Family members □ Friends

**2. Why are you willing to get a flu vaccine? (Multiple choices allowed)**

□ I heard there is a free policy

□ Community promotion of the benefits of vaccination

□ My family suggested getting vaccinated

□ Doctor recommended vaccination

□ Everyone around me has been vaccinated

□ I believe that vaccination can prevent diseases

□ I have previously contracted the disease and experienced its pain. To prevent future outbreaks or alleviate clinical symptoms, I want to get vaccinated.

**3. Why haven't you received the flu vaccine? (Multiple choices allowed)**

□ Haven't heard of this vaccine

□ I don't think I will get it, feel no need to vaccinate

□ Vaccination may not necessarily prevent the disease

□ Concerns about vaccine safety and potential side effects

□ Have contraindications to vaccination

□ Too expensive, can't afford it

□ It's inconvenient to get vaccinated, too troublesome
