# Supplementary material for: Impact of a Free Influenza Vaccination Policy on Older Adults in Zhejiang, China: Cross-Sectional Survey of Vaccination Willingness and Determinants
Source: JMIR Hum Factors. 2025 Sep 15;12:e73940. doi: 10.2196/73940 (PMC12435753; doi:10.2196/73940)
Supplement: Multimedia Appendix 3 [file humanfactors-v12-e73940-s003.docx]

**Multimedia Appendix 3.** Table 1

**Table S1.** Comparison of influenza vaccination willingness among different groups

| Characteristics | No. of survey, n | Influenza vaccination willingness, n (%) | | | H | *P.* |
| --- | --- | --- | --- | --- | --- | --- |
|  |  | Positive | hesitation | Negative |  |  |
| Sex |  |  |  |  | 14.53 | <.001 |
| Male | 3,566 | 2,564(71.90) | 597(16.7) | 405(11.4) |  |  |
| Female | 3,537 | 2,632(74.41) | 478(13.5) | 427(12.1) |  |  |
| Age (year) |  |  |  |  | 92.04 | <.001 |
| 60-69 | 3,472 | 2,371(68.29) | 654(18.8) | 447(12.9) |  |  |
| 70-79 | 3,061 | 2,389(78.05) | 353(11.5) | 319(10.4) |  |  |
| ≥80 | 570 | 436(76.5) | 68(12) | 66(12) |  |  |
| Marital status |  |  |  |  | 9.67 | .05 |
| Unmarried | 55 | 39(71) | 7(13) | 9(16) |  |  |
| Married | 6,108 | 4,463(73.07) | 950(15.6) | 695(11.4) |  |  |
| Unmarried/Divorced/Widowed | 940 | 694(73.8) | 118(12.6) | 128(13.6) |  |  |
| Family structure |  |  |  |  | 20.00 | .003 |
| Solitary living | 713 | 530(74.3) | 89(12) | 94(13) |  |  |
| Living with spouse or children | 4,317 | 3,196(74.03) | 648(15.0) | 473(11.0) |  |  |
| Living with spouse and children | 2,009 | 1,432(71.28) | 320(15.9) | 257(12.8) |  |  |
| Other | 64 | 38(59) | 18(28) | 8(13) |  |  |
| Education |  |  |  |  | 21.43 | .002 |
| Primary school or lower | 4,577 | 3,389(74.04) | 637(13.9) | 551(12.0) |  |  |
| Middle school | 1,615 | 1,142(70.71) | 275(17.0) | 198(12.3) |  |  |
| High school/Technical school | 723 | 525(72.6) | 129(17.8) | 69(10) |  |  |
| College or higher | 188 | 140(74.5) | 34(18) | 14(7) |  |  |
| Occupation |  |  |  |  | 61.20 | <.001 |
| Agency/ Institutional personnel | 376 | 293(77.9) | 51(14) | 32(9) |  |  |
| Medical staff | 215 | 176(81.9) | 28(13) | 11(5) |  |  |
| Farmers | 3,652 | 2,763(75.66) | 502(13.8) | 387(10.6) |  |  |
| Corporate staff | 951 | 671(70.6) | 149(15.7) | 131(13.8) |  |  |
| Sole proprietors | 723 | 485(67.1) | 136(18.8) | 102(14.1) |  |  |
| Other | 1,186 | 808(68.1) | 209(17.6) | 169(14.3) |  |  |
| Income (RMB) |  |  |  |  | 17.79 | .02 |
| <2000 | 2,653 | 2,004(75.54) | 356(13.4) | 293(11.0) |  |  |
| 2000-4999 | 3,316 | 2,372(71.53) | 530(16.0) | 414(12.5) |  |  |
| 5000-10000 | 743 | 542(73.0) | 117(15.8) | 84(11) |  |  |
| >10000 | 80 | 57(71) | 17(21) | 6(8) |  |  |
| Unclear | 311 | 221(71.1) | 55(18) | 35(11) |  |  |
| Chronic illness status |  |  |  |  | 34.91 | <.001 |
| None | 1,931 | 1,355(70.17) | 365(18.9) | 211(10.9) |  |  |
| 1 to 2 | 4,545 | 3,377(74.30) | 637(14.0) | 531(11.7) |  |  |
| 3 or more | 627 | 464(74.0) | 73(12) | 90(14) |  |  |
| Influenza  vaccination  history |  |  |  |  | 926.70 | <.001 |
| Yes | 4,121 | 3,572(86.68) | 347(8.4) | 202(4.9) |  |  |
| No | 2,982 | 1,624(54.46) | 728(24.4) | 630(21.1) |  |  |
