# Supplementary material for: Impact of a Free Influenza Vaccination Policy on Older Adults in Zhejiang, China: Cross-Sectional Survey of Vaccination Willingness and Determinants
Source: JMIR Hum Factors. 2025 Sep 15;12:e73940. doi: 10.2196/73940 (PMC12435753; doi:10.2196/73940)
Supplement: Multimedia Appendix 4 [file humanfactors-v12-e73940-s004.docx]

**Multimedia Appendix 4.** Table 4

**Table S4.** The means of obtaining health information among different age groups of participants

| Ways of acquisition | No. of selected | Age ,n(%) | | | H | *P.* |
| --- | --- | --- | --- | --- | --- | --- |
|  |  | 60-69 | 70-79 | ≥80 |  |  |
| Television | 4,446 | 2,242(64.57) | 1,885(61.58) | 319(56.0) | 17.85 | <.001 |
| Radio | 2,082 | 1,040(29.95) | 903(29.5) | 139(24.4) | 7.42 | .02 |
| newspapers/magazines | 1,484 | 769(22.2) | 618(20.2) | 97(17) | 9.41 | .01 |
| Websites | 671 | 421(12.1) | 217(7.1) | 33(6) | 57.93 | <.001 |
| WeChat | 2,078 | 1,366(39.34) | 634(20.7) | 78(14) | 345.44 | <.001 |
| TikTok | 1,368 | 948(27.3) | 384(12.5) | 36(6) | 294.66 | <.001 |
| Doctors | 4,143 | 2,006(57.78) | 1,805(58.97) | 332(58.3) | .95 | .62 |
| Family members | 3,675 | 1,703(49.05) | 1,638(53.51) | 334(58.6) | 24.65 | <.001 |
| Friends | 2,392 | 1,226(35.31) | 1,007(32.90) | 159(27.9) | 13.52 | <.001 |
