# Supplementary material for: Impact of a Free Influenza Vaccination Policy on Older Adults in Zhejiang, China: Cross-Sectional Survey of Vaccination Willingness and Determinants
Source: JMIR Hum Factors. 2025 Sep 15;12:e73940. doi: 10.2196/73940 (PMC12435753; doi:10.2196/73940)
Supplement: Multimedia Appendix 5 [file humanfactors-v12-e73940-s005.docx]

**Multimedia Appendix 5.** Table 5

**Table S5.** Comparison of the reasons for willingness to receive influenza vaccination among different age groups

| Reasons | No. of selected | Age, n(%) | | | H | *P.* |
| --- | --- | --- | --- | --- | --- | --- |
|  |  | 60-69 | 70-79 | ≥80 |  |  |
| Awareness of the free vaccine policy | 2,869 | 867(61.9) | 1,703(73.98) | 299(72.2) | 61.25 | <.001 |
| Active promotion by the community | 2,171 | 739(52.8) | 1,238(53.78) | 194(46.9) | 6.74 | .03 |
| Advice from family Members | 1,594 | 568(40.5) | 873(37.9) | 153(37.0) | 3.12 | .21 |
| Advice from medical staff | 1,730 | 606(43.3) | 959(41.7) | 165(39.9) | 1.78 | .41 |
| Influence of vaccination among surrounding persons | 1,111 | 378(27.0) | 628(27.3) | 105(25.4) | .66 | .72 |
| Awareness of the advantages of getting vaccinated | 1,224 | 488(34.8) | 646(28.1) | 90(22) | 33.13 | <.001 |
| Awareness of the downsides of influenza infection | 264 | 102(7.3) | 140(6.1) | 22(5) | 3.04 | .22 |
