# Supplementary material for: Impact of a Free Influenza Vaccination Policy on Older Adults in Zhejiang, China: Cross-Sectional Survey of Vaccination Willingness and Determinants
Source: JMIR Hum Factors. 2025 Sep 15;12:e73940. doi: 10.2196/73940 (PMC12435753; doi:10.2196/73940)
Supplement: Multimedia Appendix 6 [file humanfactors-v12-e73940-s006.docx]

**Multimedia Appendix 6.** Table 6

**Table S6.** Comparison of the reasons for unwillingness to receive influenza vaccination among different age groups

| Reasons | No. of selected | Age, n(%) | | | H | *P.* |
| --- | --- | --- | --- | --- | --- | --- |
|  |  | 60-69 | 70-79 | ≥80 |  |  |
| Not previously acknowledged or recognized | 706 | 482(24.2) | 188(25.4) | 36(24) | .46 | .80 |
| Concerns about the necessity of vaccination | 1,411 | 993(49.9) | 345(46.5) | 73(48) | 2.57 | .28 |
| Concerns about the effectiveness of vaccines | 655 | 464(23.4) | 150(20.4) | 41(27) | 4.60 | .10 |
| Concerns regarding vaccine safety | 476 | 300(15.1) | 142(19.2) | 34(23) | 10.96 | .004 |
| Vaccination contraindicated due to existing conditions | 170 | 91(5) | 73(10) | 6(4) | 28.40 | <.001 |
| Concerns about the affordability of vaccination | 206 | 158(8.0) | 37(5) | 11(7) | 6.96 | .03 |
| Concerns about the accessibility of vaccines | 442 | 311(15.7) | 102(13.8) | 29(19) | 3.29 | .19 |
